# Nanomaterial‐Assisted Photodynamic and Photothermal Therapy for Acne Vulgaris: A Potential Strategy for Enhancing Therapeutic Efficacy

**DOI:** 10.1111/jocd.71163

**Published:** 2026-09-07

**Authors:** Shiqian Lei, Yuting Meng, Chunbo Yu, Nana Sun, Yun Liu

**Affiliations:** ^1^ The Key Laboratory of Pharmaceutical Research for Tumor Prevention and Treatment of the Education Department of Guizhou Province Zunyi Medical University Zunyi China; ^2^ Department of Dermatology Guizhou Province Cosmetic Plastic Surgery Hospital, Affiliated Hospital of Zunyi Medical University Zunyi China

**Keywords:** acne vulgaris, combination therapy, follicular delivery, nanomaterials, photodynamic therapy, photothermal therapy

## Abstract

**Background:**

Photodynamic therapy (PDT) and photothermal therapy (PTT) showed promising efficacy for acne vulgaris in recent studies. Nevertheless, their therapeutic efficacy for acne vulgaris may be restricted by limited light penetration and insufficient accumulation of photosensitizers or photothermal agents within the pilosebaceous unit. Nanomaterial‐assisted strategy may help overcome limitations and enhance the efficacy of acne vulgaris PDT and PTT. This strategy can assist acne vulgaris PDT and PTT in follicular delivery, local light capture, and optical energy conversion, and support combination therapies that integrate PDT, PTT, and other treatment approaches. This review summarizes studies on nanomaterial‐assisted PDT and PTT for acne vulgaris, systematically discussing the potential mechanisms by which nanomaterials enhance their therapeutic efficacy.

**Aim:**

This review aims to elucidate how nanomaterials enhance PDT and PTT efficacy in acne vulgaris and to show their potential as novel therapeutic strategies for acne vulgaris.

**Methods:**

The PubMed database was searched using terms including “acne vulgaris,” “PDT,” “PTT,” and “nanomaterials,” focusing on the mechanisms and therapeutic efficacy of nanomaterial‐assisted PDT and PTT for acne vulgaris.

**Results:**

Given the limited database and search scope, this review provides a non‐exhaustive synthesis of current evidence. Preliminary findings suggest that nanomaterials can enhance the therapeutic efficacy of PDT and PTT for acne vulgaris. However, the evidence is largely limited to preclinical and small, single‐arm clinical studies.

**Conclusions:**

Nanomaterial‐assisted PDT and PTT represent promising therapeutic approaches for acne vulgaris. Further clinical trials are needed to facilitate their clinical translation.

## 1 Introduction

Phototherapies for acne vulgaris are physical treatment modalities with favorable patient adherence, among which photodynamic therapy (PDT) and photothermal therapy (PTT) attracted increasing attention in recent years [1, 2, 3]. Acne vulgaris PDT relies on light to activate photosensitizers to generate reactive oxygen species (ROS). These ROS can kill acne‐associated *Cutibacterium acnes* (*C. acnes*) and modulate inflammatory responses driven by abnormal sebum secretion [4]. The light sources used in PDT include visible red light, blue light, intense pulsed light (IPL), certain lasers [5]. Clinical trials have demonstrated the therapeutic efficacy of acne vulgaris PDT [6]. Acne vulgaris PTT, also referred to as selective sebaceous gland photothermolysis in dermatology, includes 1726‐nm laser treatment and nanoparticle‐assisted laser therapy [2]. This approach mainly utilizes high‐energy laser irradiation, either directly or with nanoparticle assistance, to induce heat generation within the pilosebaceous unit. The generated thermal energy can kill *C. acnes* and disrupt abnormal sebaceous gland structures [7, 8]. The efficacy of these therapies has been confirmed in recent clinical trials, suggesting that they are promising physical treatment strategies for acne vulgaris.

**FIGURE 1:**
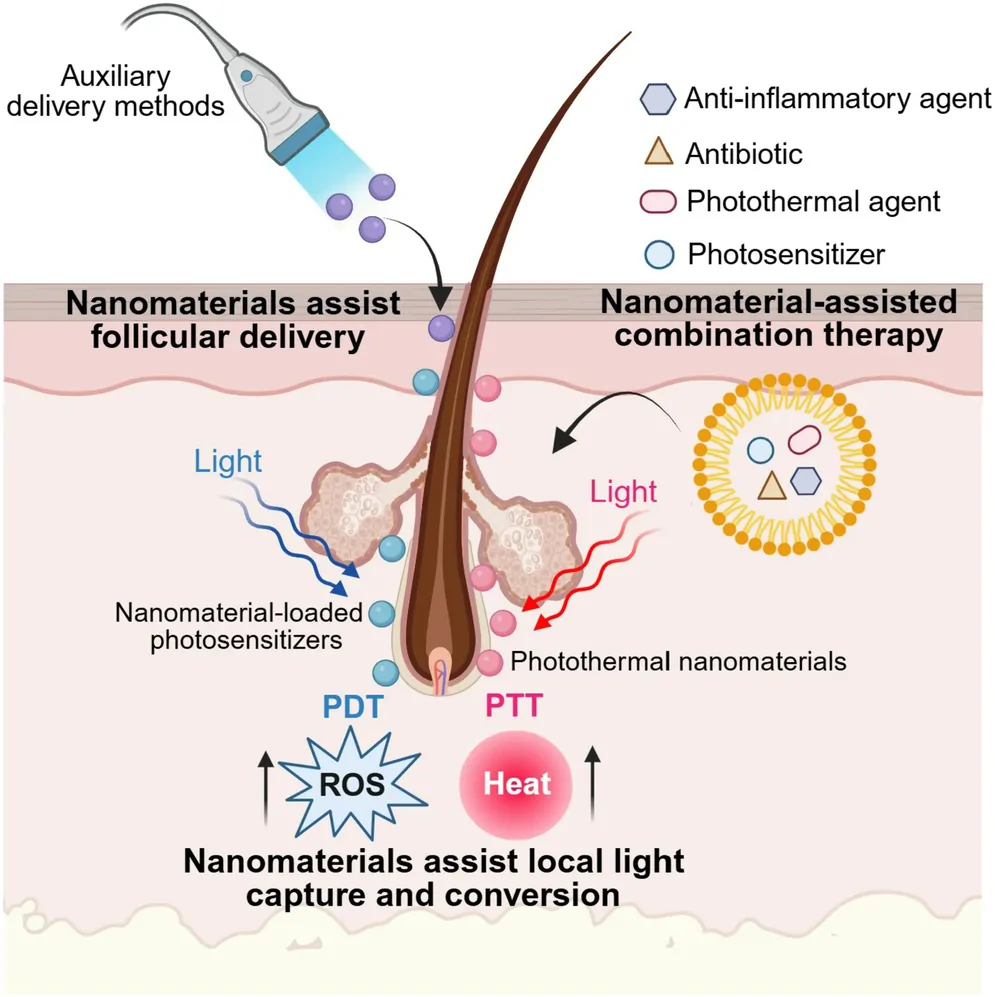
Potential mechanisms of nanomaterial‐assisted acne vulgaris PDT and PTT for enhancing therapeutic efficacy: Nanomaterials can carry photosensitizers or act as photothermal agents to enable more precise delivery to the pilosebaceous unit. After delivery to the pilosebaceous unit, nanomaterials can enhance local light capture and assist the conversion of optical energy into ROS or heat. Nanomaterials can also serve as combination platforms for the simultaneous loading of photosensitizers, photothermal agents, and other therapeutic agents, thereby enabling integrated management of acne vulgaris. Created in BioRender. Lei, S. (2026) https://BioRender.com/27nkpcc.

Although PDT and PTT have shown favorable efficacy in some clinical trials for acne vulgaris, they cannot replace topical or systemic pharmacological therapy [9]. Ishii et al. [10] reported that the efficacy of acne vulgaris PDT and PTT did not differ significantly from that of commonly used anti‐acne medications. The therapeutic effects of PDT and PTT may be limited by insufficient light penetration, inadequate distribution of photosensitizers or photothermal agents within the pilosebaceous unit, and other related factors [11, 12]. The use of nanomaterials may help overcome these limitations and improve the efficacy of acne vulgaris PDT and PTT [13, 14]. Nanomaterials represent an important emerging adjunctive strategy for acne vulgaris treatment [15]. Due to their unique physicochemical properties, nanomaterials can assist the follicular delivery of photosensitizers and photothermal agents, promote their accumulation within the pilosebaceous unit, and assist local light capture and energy conversion at the treatment area [16, 17]. In addition, nanomaterials can assist the combination of PDT, PTT, and other therapeutic modalities, thereby enabling integrated management of acne vulgaris [18, 19]. These three key mechanisms are illustrated in Figure 1. Based on these mechanisms, nanomaterial‐assisted strategies may improve the therapeutic efficacy for acne vulgaris PDT and PTT.

Previous reviews have mainly focused on summarizing the efficacy and safety of acne vulgaris PDT and PTT [20]. Regarding nanomaterials, existing reviews have primarily discussed their application in topical drug treatment of acne vulgaris [21, 22]. This review focuses on studies investigating nanomaterial‐assisted acne vulgaris PDT and PTT and discusses the potential therapeutic enhancement provided by this strategy. We hope that this review will offer new perspectives and insights for the clinical application of acne vulgaris PDT and PTT.

## 2 Discussion

### 2.1 Nanomaterials Assist Follicular Delivery

The pathophysiological of acne vulgaris is widely believed to be associated with the pilosebaceous unit, involving the follicular opening, follicular infundibulum, sebaceous gland, and the local *C. acnes*‐rich microenvironment [23]. Therefore, Latter et al. [24] summarized that topical therapeutic efficacy for acne vulgaris largely depends on whether the active agent can reach the pilosebaceous unit. However, the stratum corneum limits the follicular delivery efficiency of topical treatments and may contribute to local irritation and side effects [25]. Combining nanomaterials for acne vulgaris treatment can assist follicular delivery and overcom these limitations [26, 27]. Castro et al. [28] used solid lipid nanoparticles to encapsulate all‐trans retinoic acid, thereby alleviating retinoic acid‐induced skin irritation. The assisted effect is believed to be related to properties of nanomaterials. Lademann et al. [29] demonstrated that a dye in nanoparticle form is more easily delivered through the appendage pathway into the hair follicle compared to non‐particulate forms. Patzelt et al. [30] also found that this follicular delivery can be regulated by adjusting the particle size of the nanomaterials. The results showed that nanoparticles of different sizes affect the depth of penetration into the pilosebaceous unit. In 2015, Svenskaya et al. [31] summarized the mechanisms of follicular drug delivery using nanoparticulate systems, including the hair shaft‐driven “ratchet effect” and interactions with sebum etc. They further indicated that optical methods, including fluorescence imaging and Raman spectroscopy, can be used to evaluate the efficiency of follicular delivery of particulate systems. Based on these mechanisms, nanomaterials can also assist photosensitizers and photothermal agents in follicular delivery for acne vulgaris PDT and PTT.

**FIGURE 2:**
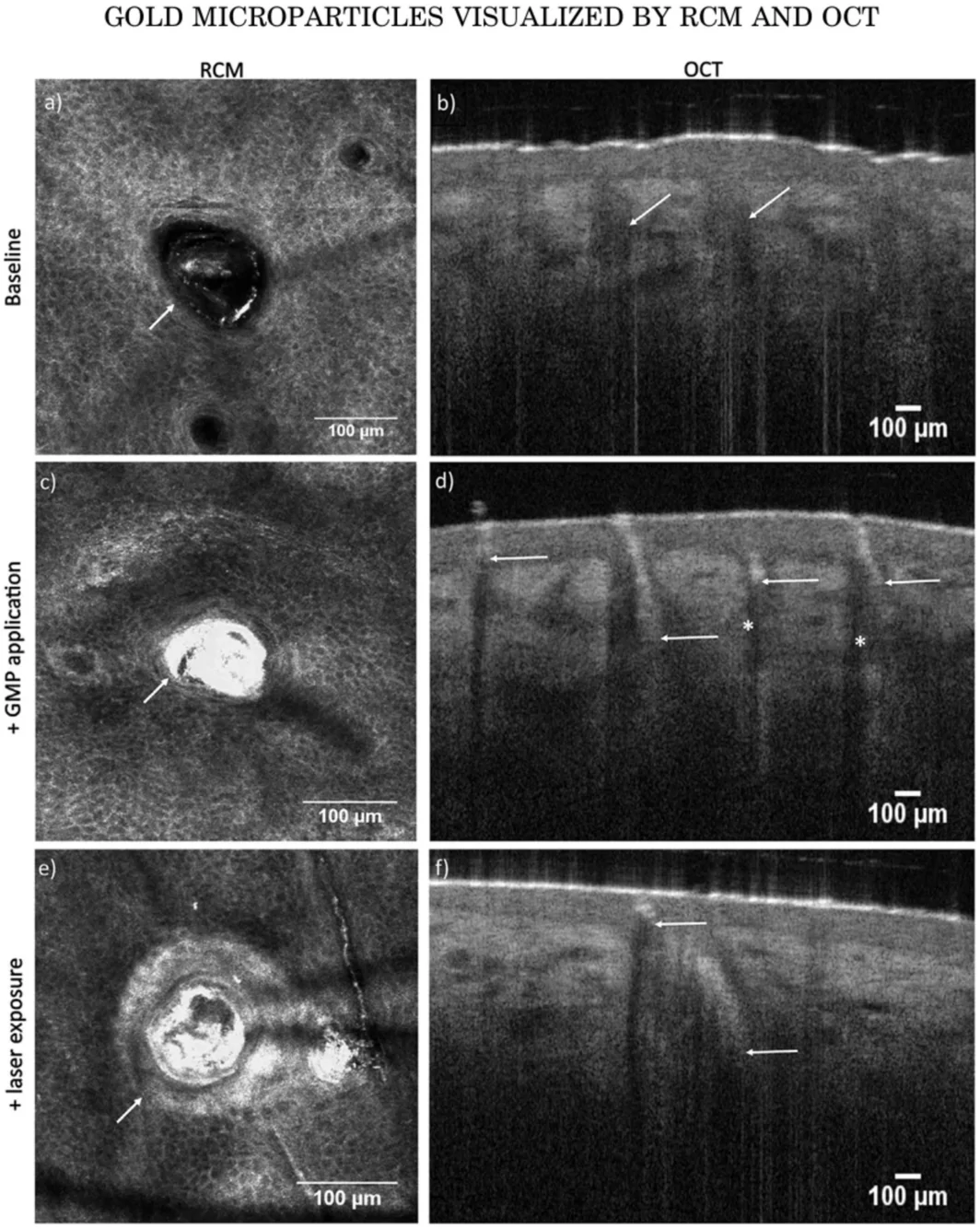
RCM and OCT images showing follicular delivery at baseline, after gold microparticle application and laser exposure. Adapted from Fuchs et al. [35] with permission from Wiley Periodicals Inc.

For acne vulgaris PDT, nanomaterials can assist the delivery of photosensitizers. Fadel et al. [32] used liposomes to load methylene blue (MB). They compared the effects of liposomes loaded with MB to those of free MB in mice. The results showed that free MB gel caused surface damage only in mouse fur, while liposomal MB showed complete destruction of the pilosebaceous unit. These intuitive results indicate that these nanomaterials can achieve targeted treatment in the pilosebaceous unit. Furthermore, this assistive delivery role of nanomaterials for photosensitizers can also reduce the side effects of PDT. Hongcharu et al. [33] showed that side effects of 5‐aminolevulinic acid‐mediated PDT (ALA‐PDT) result from ALA deposited in the normal tissue, releasing ROS to induce an inflammatory response and trigger pain. Nanomaterials lower the required ALA concentration to reduce side effects by assisting ALA follicular delivery. Leeuw et al. [34] used a 5‐ALA 0.5% liposomal spray and IPL in combination with topical keratolytic agents for acne vulgaris treatment. Compared to PDT with a 20% 5‐ALA cream, the 5‐ALA concentration was reduced 40‐fold and the treatment showed fewer side effects. These experiments have demonstrated that using nanomaterials to assist acne vulgaris PDT is a promising treatment approach. For acne vulgaris PTT, gold‐based light‐absorbing nanoparticles are often used as photothermal agents. These gold nanoparticles are more easily deposited into the hair follicle and act in the pilosebaceous unit. Fuchs et al. [35] assessed the follicular delivery of gold microparticles (GMPs) in acne vulgaris skin by reflectance confocal microscopy (RCM) and optical coherence tomography (OCT). In RCM images, GMPs appeared highly reflective within the pilosebaceous unit. In OCT images, GMPs also appeared highly reflective within hair follicles, while they were not detected in the surrounding skin. The results are shown in Figure 2. This evidence directly shows that nanomaterials can assist the follicular delivery for acne vulgaris PTT. In current studies of acne vulgaris PTT, nanomaterials are also combined with additional delivery methods. This combined approach demonstrates higher follicular delivery efficiency. For example, Paithankar et al. [7] used mechanical vibration to deliver gold‐coated silica microparticles into the pilosebaceous unit of human and swine in vivo. The results showed better follicular delivery efficiency than not using mechanical vibration. This approach appears to be an effective treatment for acne vulgaris.

Nanomaterials can assist the follicular delivery of photosensitizers and photothermal agents, thereby potentially improving therapeutic efficacy and reducing side effects of acne vulgaris PDT and PTT. However, these positive trial results are largely limited to animal and in vitro studies. Some studies also suggest that nanomaterials are not retained sufficiently well within the pilosebaceous unit, which may limit the long‐term efficacy of this adjunctive therapy [18]. Further research is needed to validate the use of nanomaterials to facilitate follicular delivery for acne vulgaris PDT and PTT and to address possible potential limitations.

### 2.2 Nanomaterials Assist Local Light Capture and Conversion

Skin tissue has optical properties that absorb and scatter light. Setchfield et al. [36] summarized different components in the skin tissue, such as blood, water, melanin, all of which have different properties that absorb or scatter light. These optical properties of skin tissue cause the actual effective light energy received in the pilosebaceous unit to be lower than the external irradiation dose, which may limit the capture efficacy of light. For example, Uzunbajakava et al. [37] clearly pointed out that the penetration depth of blue light used to treat acne vulgaris is poor and will be lost due to scattering or melanin absorption. A classical strategy to overcome this limitation is the use of optical clearing agents (OCA) to reduce refractive index among different skin components, thereby decreasing light scattering. For example, Chu et al. [38] formulated a skin optical clearing gel using glycerol as the principal component in combination with oleic acid as a skin penetration enhancer. After treatment with this OCA, the transmittance of light through porcine skin increased to approximately 70%, compared with only approximately 10% without OCA treatment. In recent research, combining nanomaterials to mitigate this limitation has rapidly developed. Loiseau et al. [39] reported that silver nanomaterials can strongly absorb light at specific wavelengths through localized surface plasmon resonance. For acne vulgaris PTT, when nanomaterials with strong light absorption properties are enriched in the pilosebaceous unit, they can serve as exogenous light absorption centers, increasing the effective optical absorption of the target region. Fuchs et al. [40] evaluated the efficacy of light‐absorbing gold nanoparticles and pulsed light therapy for severe acne vulgaris patients. The results suggest that this treatment approach shows high promise for the treatment of acne vulgaris. For acne vulgaris PDT, nanomaterials can also serve as auxiliary delivery systems to increase the levels of light‐absorbing substances in the treatment area, thereby achieving enhanced local light capture. Moftah et al. [41] compared the efficacy of PDT with liposomal methylene blue (LMB) and the use of IPL alone for acne vulgaris treatment. Results showed that LMB–IPL was more effective than IPL alone for back acne vulgaris treatment. These experiments suggest that the use of nanomaterials can assist in local light capture during acne vulgaris PTT and PDT.

**FIGURE 3:**
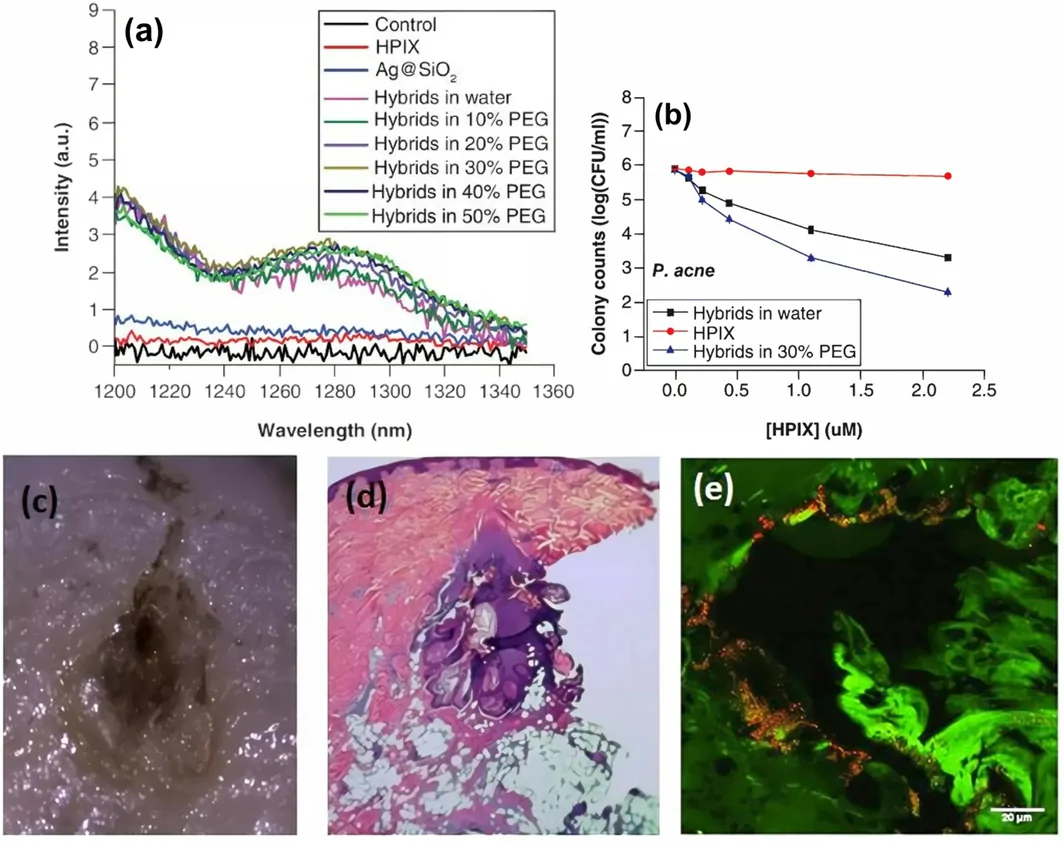
Nanomaterials enhance the phototherapeutic energy conversion for acne vulgaris PDT and PTT. (a) Singlet oxygen emission spectra of Ag@mSiO_2_@HPIX nanoparticles under 400 nm excitation. (b) Colony counts of *P. acnes* (*Propionibacterium acnes*, also known as *Cutibacterium acnes*) under illumination with HPIX or Ag@mSiO_2_@HPIX nanoparticles. Panels (a) and (b) are adapted from Wang et al. [43], with permission from Taylor & Francis. (c–e) Images showing silica‐gold nanoshell‐mediated photothermolysis therapy of the pilosebaceous unit in porcine skin. Panels (c–e) are adapted from Paithankar et al. [45], with permission from Elsevier.

Nanomaterials also provide a method for enhancing local phototherapeutic energy conversion. For acne vulgaris PDT, the therapeutic effects primarily rely on the photosensitizers to convert light of specific wavelength into ROS. The ROS generated by photoactivated photosensitizers can directly kill *C. acnes*. They can also induce damage to abnormally active sebaceous gland cells, reducing sebum‐induced inflammatory stimulation [4]. Nanomaterials can stabilize photosensitizers in PDT or serve as photocatalytic platforms to assist the efficiency of light energy conversion into ROS. For example, Wen et al. [42] improved the photostability of photosensitizer indocyanine green through loading it into the zeolitic imidazolate framework‐8. Wang et al. [43] developed nanoparticle‐based photosensitizers containing a silver core and a mesoporous silica shell with hematoporphyrin IX (Ag@mSiO_2_@HPIX) embedded for acne vulgaris treatment. After 410 nm LED irradiation, the hybrid photosensitizers showed higher levels of ROS and stronger killing activity against *C. acnes*. Results are shown in Figure 3a,b. These intuitive findings suggest that nanomaterial‐based photosensitizing systems can enhance the photoenergy conversion efficiency for acne vulgaris PDT. For acne vulgaris PTT, photothermal nanomaterials can absorb incident local light and dissipate the absorbed energy as heat through rapid non‐radiative relaxation. Cui et al. [44] reviewed the relevant mechanisms of photothermal energy conversion by photothermal nanomaterials and their recent progress. The heat released by photothermal nanomaterials can cause selective thermal injury or disruption of sebaceous gland structures, achieving treatment for acne vulgaris. Paithankar et al. [45] used ultrasound to deliver silica‐gold nanoshells for photothermolysis therapy of the pilosebaceous unit in porcine skin. The results demonstrated that silica‐gold nanoshells could selectively induce photothermal disruption of the pilosebaceous unit while sparing the surrounding skin, as shown in Figure 3c–e. Depositing photothermal nanomaterials within the pilosebaceous units provides a method for enhancing local light‐to‐heat conversion for acne vulgaris PTT. These experiments demonstrate that using nanomaterials can potentially enhance local phototherapeutic energy conversion for acne vulgaris PDT and PTT.

Using nanomaterials can assist local light capture and enhance energy conversion for acne vulgaris PTT and PDT. Moreover, Tuchin et al. [46] proposed the combination of nanomaterials and OCA as a promising emerging strategy for further enhancing local light capture. This effect may arise because facilitated‐delivery OCA can promote the penetration and localization of nanomaterials within target tissues. This assistance strategy provides a possible way to enhance the efficacy of acne vulgaris PTT and PDT. In the future, more experimental data are needed to verify the feasibility of this strategy and to investigate its potential side effects. This assisted strategy remains far from routine clinical application.

### 2.3 Nanomaterial‐Assisted Combination Therapy and Integrated Management

Acne vulgaris is a multifactorial inflammatory disease of the pilosebaceous unit. Vasam et al. [47] systematically summarized the four major mechanisms of acne vulgaris occurrence: increased sebum secretion, excessive keratinization of hair follicle sebaceous units, proliferation of *C. acnes*, and inflammatory responses. Using a single therapy modality may be insufficient to simultaneously address the multiple pathogenic mechanisms of acne vulgaris. Reynolds et al. [48] suggest combining local therapies that have multiple mechanisms of action for acne vulgaris treatment. Nanomaterials can provide a comprehensive platform for the integrated management of acne vulgaris. For example, Eroğlu et al. [49] used liposomes to encapsulate tetracycline hydrochloride and retinoic acid for combined acne vulgaris treatment. The results show excellent efficacy for acne vulgaris. This co‐delivery concept using nanomaterials can extend to acne vulgaris PDT and PTT. Nanomaterials can integrate a photosensitizer or photothermal agent with non‐photoactive therapeutic agents in a system. This integration method represents a promising acne vulgaris treatment approach.

**FIGURE 4:**
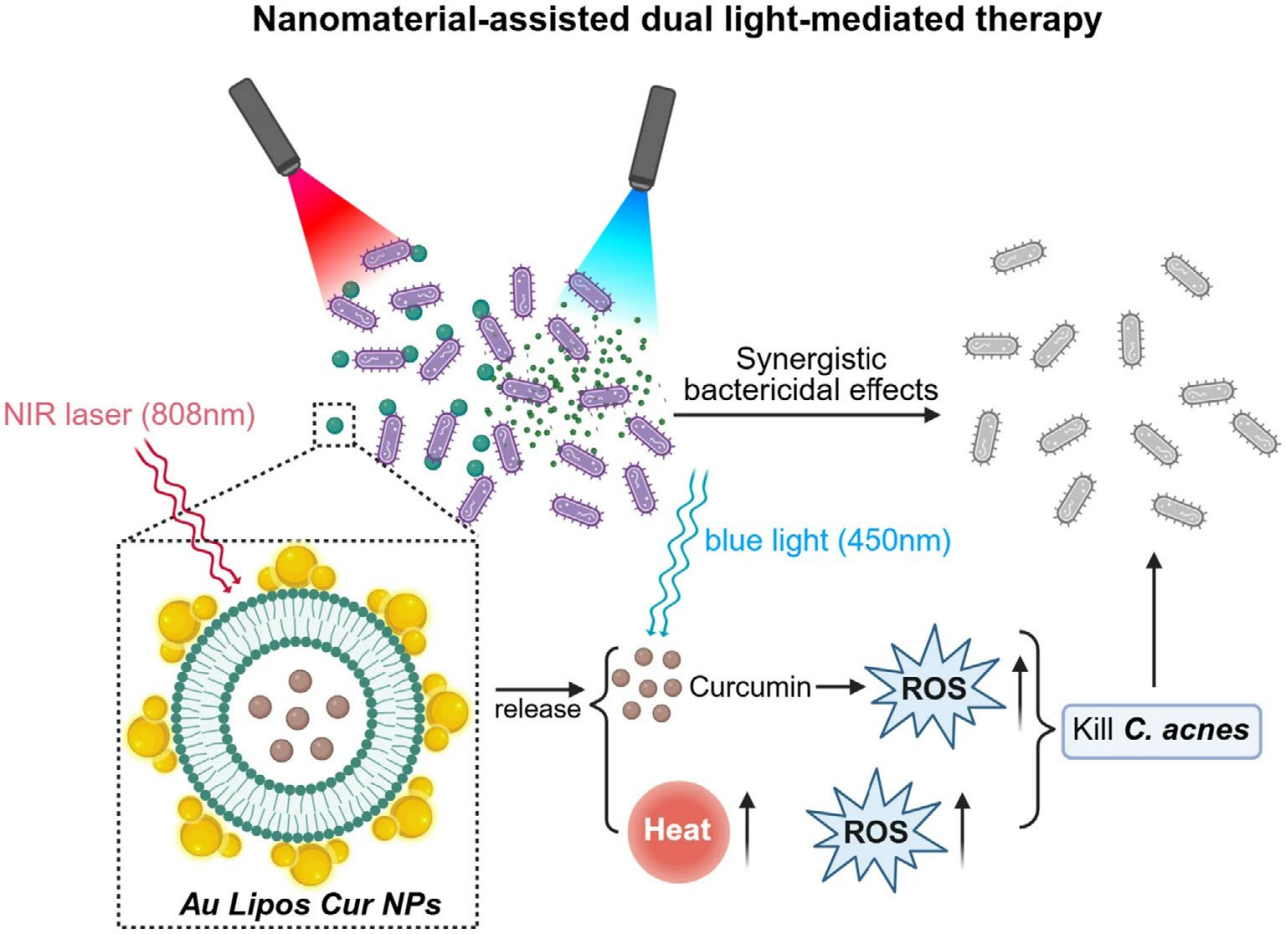
Mechanism of nanomaterial‐assisted dual light‐mediated therapy: After irradiation with an 808 nm NIR laser, the gold particles on the surface of Au Lipos Cur NPs convert light energy into heat, ROS, and release the loaded curcumin. Curcumin can act as a photosensitizer and be subsequently activated by 450 nm blue light to generate ROS. The thermal energy and ROS can synergistically kill *C. acnes* in acne vulgaris treatment. Created in BioRender. Lei, S. (2026) https://BioRender.com/vygnq4x.

For acne vulgaris PDT, using nanomaterials to simultaneously load photosensitizers and antibiotics can deliver better antibacterial and anti‐inflammatory effects. Hsiao et al. [50] developed rifampicin (RIF)‐indocyanine green‐loaded perfluorocarbon nanodroplets to provide combined photo‐, chemo‐, and probiotic efficacies to *C. acnes*. The results showed that microbial mortality was 16‐fold higher than that using loaded RIF alone. Jeong et al. [51] developed a lipase‐sensitive singlet oxygen‐producing and erythromycin‐loaded liposome (LSSPL) to achieve better inhibition of *C. acnes* growth. The *C. acnes*‐induced inflammatory infection on the back skin of nude mice was also highly decreased. These stronger antibacterial effects come from the synergism between antibiotics and PDT, and the anti‐inflammatory effects are believed to be related to the ROS produced by PDT. For acne vulgaris PTT, combining photothermal agents and non‐photoactive antimicrobial agents via nanomaterials also has high potential. This combined approach can simultaneously integrate the photothermal effect and antibacterial capability to synergistically treat acne vulgaris. Ganeshan et al. [52] developed a near‐infrared light‐absorbing copper sulfide (CuS) nanoparticle loaded with Glycyrrhizin (Ga). The photothermal efficacy studies and in vitro and in vivo experiments indicated that the mild photothermal effect mediated by CuS NPs and the bactericidal effect of Ga exerted a synergistic lethal effect on *C. acnes*. These studies suggest that the use of nanomaterials can assist in establishing a combined therapeutic platform for acne vulgaris PTT. Recent studies have also shown that nanomaterials can simultaneously integrate acne vulgaris PDT and PTT. This combination approach integrates powerful antimicrobial activity, skin regeneration capabilities, and enhanced biosafety, having significant potential for acne vulgaris treatment. For example, He et al. [19] developed a copper‐doped prussian blue (CuPB) hyaluronic acid (HA) microneedle (Cu10PB‐HA@MNs). Results showed that Cu^2+^ could enhance PB's plasmon resonance and could also generate ROS. The optimized PTT/PDT system showed dual antibacterial activity. Other compounds such as HA, Cu and Fe ions released by Cu10PB‐HA@MNs can also stimulate collagen deposition, accelerating skin regeneration. Similarly, Alvi et al. [18] developed a liposomal gold nanoparticle loaded with curcumin (Au Lipos Cur NPs) for dual light‐mediated therapy for acne vulgaris treatment. Near‐infrared irradiation first activates gold nanoparticle‐mediated photothermal and photodynamic conversion and releases curcumin. Blue light then activates curcumin‐mediated photodynamic antibacterial effects. Their treatment mechanisms are shown in Figure 4. Results showed significant inhibition of bacterial growth. These trials have demonstrated that this combination approach holds great promise for acne vulgaris treatment.

Using nanomaterials to assist acne vulgaris PDT and PTT for the combined treatment and integrated management represents a promising therapeutic strategy, possibly enhancing therapeutic effects. However, at present, this combined approach has only demonstrated efficacy in basic research, and these findings do not necessarily reflect its effectiveness in clinical trials. Meanwhile, because the interactions among the different therapies remain unclear, the potential side effects of this combined treatment cannot yet be determined. Therefore, further studies are needed to validate its therapeutic efficacy and clarify its possible side effects.

## 3 Summary and Outlook

Promising strategies have recently emerged in the treatment of acne vulgaris. Nanomaterial‐assisted acne vulgaris PDT and PTT may improve therapeutic efficacy and reduce side effects through assisting follicular delivery, local light capture and conversion, and combination therapy. We conducted a comprehensive search of PubMed using major terms including “Acne Vulgaris,” “Nanomaterials,” “Photodynamic Therapy,” and “Photothermolysis.” A total of 10 studies on nanomaterial‐assisted PDT and 17 studies on nanomaterial‐assisted PTT were identified. Most studies on nanomaterial‐assisted acne vulgaris PDT focused on liposome‐loaded photosensitizers. Studies on nanomaterial‐assisted acne vulgaris PTT primarily focused on the application of gold‐based nanostructures, with platinum‐based nanostructures also being explored. These studies, which covered in vitro experiments, animal studies, and human studies, collectively showed favorable therapeutic effects. Human studies of nanomaterial‐assisted PDT and PTT for acne vulgaris are summarized in Tables 1 and 2.

**TABLE 1:** Summary of human studies of nanomaterial‐assisted acne vulgaris PDT published on PubMed by July 14, 2026.

References	Type of trial	Study population	Nanomaterials	Light source
Moftah et al. [41]	Comparative randomized split‐body study	35 patients with varying degrees of acne	0.1% liposomal methylene blue	Intense pulsed light
Leeuw et al. [34]	Randomized, prospective, single blind study	32 patients suffering from acne	5‐ALA 0.5% in liposomal spray	Intense pulsed light
An et al. [53]	Prospective single‐arm uncontrolled clinical study	13 Korean subjects with inflammatory acne	0.5% Liposome‐encapsulated 5‐ALA	Intense pulsed light
Lee et al. [54]	Randomized controlled pilot clinical study	18 patients with mild‐to‐moderate acne vulgaris	3% Liposome‐encapsulated 5‐ALA	Intense pulsed light
Soliman et al. [55]	Prospective non‐randomized split‐face controlled clinical study	15 patients with mild‐to‐moderate facial acne	Methylene blue nanoemulgel	Pulsed dye laser and 665‐nm diode laser
Lee et al. [56]	Pre‐post implementation study with a single‐center trial	24 patients with facial acne	Nanoformulation consisting of methylene blue and salicylic acid	663‐nm light‐emitting diode light
Yeung et al. [57]	Prospective single‐arm uncontrolled pilot clinical study	12 patients (skin types IV–V) with facial acne	Liposome‐encapsulated 0.5% 5‐ALA spray	Intense pulsed light

**TABLE 2:** Summary of human studies of nanomaterial‐assisted acne vulgaris PTT published on PubMed by July 14, 2026.

References	Type of trial	Study population	Nanomaterials	Light source
Paithankar et al. [7], Trial 1	Prospective, randomized, controlled, two‐site clinical pilot study	48 patients with moderate‐to‐severe inflammatory facial acne	Light‐absorbing gold‐coated silica microparticles	800‐nm diode laser
Paithankar et al. [7], Trial 2	Prospective, controlled, two‐site, sham‐controlled clinical pilot study	51 patients with moderate‐to‐severe inflammatory facial acne	Light‐absorbing gold‐coated silica microparticles	800‐nm diode laser
Park et al. [58]	Two‐case report	2 Asian patients with recurrent acne vulgaris	Gold nanoshells	Near‐infrared laser
Suh et al. [59]	Single‐arm, uncontrolled, and preliminary clinical study	12 Asian patients with moderate‐to‐severe acne vulgaris	Gold nanoparticles	Isolaz (Solta Medical Inc., California) 400 nm tip
Suh et al. [60]	Randomized controlled preliminary clinical study	24 patients with moderate‐to‐severe acne vulgaris	Ethosome gold nanoparticles	CO_2_ fractional laser pretreatment, long‐pulsed 1064‐nm Nd: YAG laser
Fuchs et al. [40]	Open‐label, single‐arm, uncontrolled pilot clinical study	28 patients with moderate to moderately severe facial acne	Gold microparticles	800‐nm diode laser
Paithankar et al. [45]	Three‐site, non‐randomized pilot clinical study	37 patients with facial acne	Silica‐gold nanoshells	Near‐infrared 800‐nm pulsed laser
Quezada et al. [61]	Three‐case report	3 adolescent patients with mild acne	Gold nanoparticle	Q‐Switched 1064 nm laser

These human studies demonstrated favorable clinical outcomes. For example, Moftah et al. [41] conducted a randomized split‐body study involving 35 patients with truncal acne vulgaris. One side of each patient's back was treated with 0.1% LMB followed by IPL irradiation, whereas the contralateral side received IPL alone. The combination treatment resulted in a greater reduction in inflammatory acne lesions than IPL monotherapy (56.40% vs. 34.06%). Paithankar et al. [7] treated 51 patients with nanomaterials as photothermal agents. The active treatment group showed a greater reduction in inflammatory acne lesions than the sham‐treated group (49.0% vs. 21.7%). These comparative clinical findings directly supported the therapeutic enhancement caused by nanomaterials in PDT and PTT for acne vulgaris. However, most studies of this combination strategy are still limited to small, single‐arm clinical investigations and remain at the stage of preliminary efficacy evaluation. Therefore, this possible strategy for enhancing therapeutic efficacy has not yet received sufficient attention in clinical applications. The potential adverse effects of this assistive strategy also remain unclear, and direct efficacy comparisons with non‐nanomaterial‐assisted treatment modalities are still lacking. Further studies should focus on ensuring the safety of this assisted strategy and determining whether it can provide meaningful improvements in therapeutic efficacy, thereby gradually advancing its clinical application.

Current clinical trials of acne vulgaris PDT remain focused on the development of different novel photosensitizers, while clinical trials of acne vulgaris PTT are still largely limited to various laser‐based modalities [2, 6]. We hope that this review will provide new perspectives for the clinical application of acne vulgaris PDT and PTT. The use of nanomaterial‐assisted strategies represents an emerging and potentially efficacy‐enhancing approach. We anticipate that future research will further advance the clinical translation of this assistive strategy and provide more effective therapeutic options for acne vulgaris patients.

## Author Contributions

Conceptualization: Nana Sun and Yun Liu; writing and original draft preparation: Shiqian Lei; writing – review and editing, and visualization: Shiqian Lei, Yuting Meng, Chunbo Yu, Nana Sun and Yun Liu; supervision: Nana Sun and Yun Liu. All authors have read and agreed to the published version of the manuscript.

## Funding

This study was supported by the Science and Technology Plan Project of Guizhou Province (QKHZC [2022] 292), Science and Technology Foundation of the Health Commission of Guizhou Province (gzwkj2025‐219), the innovation and entrepreneurship training program of Zunyi Medical University (2024106610851).

## Conflicts of Interest

The authors declare no conflicts of interest.

## Data Availability

The data that support the findings of this study are available from the corresponding author upon reasonable request.
